# Japanese Encephalitis Virus Disrupts Cell-Cell Junctions and Affects the Epithelial Permeability Barrier Functions

**DOI:** 10.1371/journal.pone.0069465

**Published:** 2013-07-24

**Authors:** Tanvi Agrawal, Vats Sharvani, Deepa Nair, Guruprasad R. Medigeshi

**Affiliations:** Vaccine and Infectious Disease Research Center, Translational Health Science and Technology Institute, Gurgaon, India; Utah State University, United States of America

## Abstract

Japanese encephalitis virus (JEV) is a neurotropic flavivirus, which causes viral encephalitis leading to death in about 20–30% of severely-infected people. Although JEV is known to be a neurotropic virus its replication in non-neuronal cells in peripheral tissues is likely to play a key role in viral dissemination and pathogenesis. We have investigated the effect of JEV infection on cellular junctions in a number of non-neuronal cells. We show that JEV affects the permeability barrier functions in polarized epithelial cells at later stages of infection. The levels of some of the tight and adherens junction proteins were reduced in epithelial and endothelial cells and also in hepatocytes. Despite the induction of antiviral response, barrier disruption was not mediated by secreted factors from the infected cells. Localization of tight junction protein claudin-1 was severely perturbed in JEV-infected cells and claudin-1 partially colocalized with JEV in intracellular compartments and targeted for lysosomal degradation. Expression of JEV-capsid alone significantly affected the permeability barrier functions in these cells. Our results suggest that JEV infection modulates cellular junctions in non-neuronal cells and compromises the permeability barrier of epithelial and endothelial cells which may play a role in viral dissemination in peripheral tissues.

## Introduction

Japanese encephalitis virus (JEV) is a mosquito-borne flavivirus from the family *Flaviviridae*. JEV genome comprises of a single-stranded, positive-sense RNA which encodes a single polyprotein cleaved by both the host and viral proteases to derive 3 structural (C, prM-M and E) and 7 non-structural (NS1, 2A, 2B, 3, 4A, 4B and 5) proteins. JEV is the leading cause of viral encephalitis in South and South-East Asia. There are no antivirals against JEV and non-availability of affordable vaccines in endemic areas are a major setback in combating JEV and other closely-related viruses [1]. JEV infection is initiated with the bite of an infected *Culex* mosquito and the virus presumably replicates in Langerhans cells and spreads to peripheral tissues establishing a systemic infection. Severe manifestations of JEV disease is due to viral entry into the central nervous system (CNS) triggering activation of microglia resulting in neuronal damage [2], [3]. JEV replication has been shown to occur also in extra-neural tissues in animal models when the virus is delivered via peripheral inoculation [4]. Our preliminary studies in mouse models confirm these observations. In various mouse models of JEV, the virus has been isolated from kidney, liver and spleen indicating that JEV infects peripheral tissues *in vivo* [5], [6]. However, the kinetics of virus dissemination in peripheral organs has not been characterized primarily due to lack of relevant animal models in which these aspects could be addressed. Previous studies have reported abnormalities in liver and kidney function and gastric hemorrhage in pediatric and adult patients infected with JEV [7], [8]. It is not known if these are direct effects of JEV replication in these tissues or due to host response. Other closely related viruses in the *Flaviviridae* family, including neurotropic viruses, have been shown to have broad tissue tropism both in experimental animals and humans [9]–[12]. Virus replication in epithelial and endothelial cells of peripheral tissues that form the permeability barrier may play a key role in the overall outcome of JEV infection *in vivo*. Tight junctions (TJ) play an essential role in maintaining the permeability barrier of epithelial and endothelial cells which regulates tissue homeostasis. TJ is composed of a multi-protein complex, which includes the tetraspanin claudins, occludin and the cytosolic proteins such as zona occludens (ZO), which connects the cytoskeletal assembly to the TJ membrane proteins. Intercellular TJ contacts create a fence-like barrier separating the apical and basolateral surfaces thereby regulating a number of physiological functions such as cell polarity, differentiation and proliferation. However, the diffusion barrier created by TJ is dynamic in nature and paracellular transport is regulated by the components of TJ most importantly by claudins in response to the tissue requirement for micro and macromolecular solutes. TJ also acts as functional diffusion barrier for pathogens and many viruses either use components of TJs for establishing infection in epithelial or endothelial cells or modulate the TJ components to gain access to the tissue space and for further spread of the infection [13], [14].

Although both epithelial and endothelial cells form permeability barrier *in vivo*, endothelial cells by themselves fail to form tight junctions in vitro and physiologically relevant endothelial barrier is formed in co-culture systems used to study blood-brain-barrier [15], [16]. In contrast, epithelial cell lines such as Caco-2 serve as an excellent model to study permeability barrier as they form a functional barrier which is due to the formation of tight junctions [17]. In this study, we have investigated the effect of JEV infection on cellular junctions of epithelial cells (Caco-2), hepatocytes (Huh7) and primary endothelial cells (HUVEC). We show that JEV infection affects cell-cell junctions in all these cell lines. JEV affected the barrier functions at later stages of infection by causing degradation of a number of TJ and adherens junction (AJ) proteins in all the cell lines tested. We found robust induction of genes involved in antiviral response, cellular stress response and generation of reactive oxygen species (ROS) upon JEV infection in epithelial cells. However, we did not detect induction of cytokines or matrix metalloproteinases (MMP) in infected culture supernatants of epithelial cells whereas a strong inflammatory response was observed in endothelial cells infected with JEV. Treating infected epithelial cells with bafilomycin A1 (an inhibitor of vacuolar ATPase proton pump thus preventing endosome/lysosome acidification and activation of lysosomal enzymes) was able to rescue the loss of claudin-1 suggesting the involvement of lysosomal degradation. Finally, Caco-2 clones expressing the JEV-capsid protein were capable of significantly affecting the barrier functions. We observed a significant increase in viral titers in these clones suggesting that the disruption of barrier functions may enhance release of infectious virus particles. Based on these results, we propose an important role for cellular junction disruption in viral dissemination in peripheral organs thus influencing the outcome of the disease caused by JEV infection.

## Materials and Methods

### Cells and Viruses

All cell lines were obtained from National Center for Cell Science, Pune India. Caco-2 and Huh7 cells were propagated in Dulbecco’s modified Eagle medium (DMEM) (Invitrogen) supplemented with 10% fetal bovine serum (FBS), 2 mM L-glutamine (Invitrogen), 100 units/ml penicillin G sodium and 100 µg/ml streptomycin sulfate (Invitrogen) and non-essential amino acids. Porcine kidney (PS) cells were grown in the minimal essential medium (MEM) containing 10% FBS, Earle’s salts and additives as mentioned above. Human umbilical vein endothelial cells (HUVEC) were purchased from BD Biosciences and grown in endothelial cell culture medium supplied with the cell line. JEV-Vellore strain was used throughout the study and viral titers were estimated as described previously [18]. For generation of virus-free culture supernatants, Caco2 cells were infected with JEV at an MOI of 5 pfu/cell and at around 44 hour post-infection (h pi) supernatants were collected and clarified through Amicon columns with 100 k Da cut-off (Merck-Millipore). Clarified supernatants were used for further experiments. No virus could be detected in the clarified supernatants by plaque assay.

### Construction of Plasmids and Transfection

The capsid encoding region without the trans-membrane region was amplified from viral RNA using Titan one-step RT-PCR kit (Roche) using the following forward and reverse primers: 5′-tacatatgactaaaaaaccaggag-3′ and 5′-gactcgagtcttttgttttgttttctgcc-3′. JEV-C fragment was cloned into pET23b as a NdeI – XhoI fragment and transformed into E. coli strain BL21DE3-pLys-S and recombinant capsid with a c-terminal His-tag was expressed and purified using Nickel-NTA beads as per the manufacturer’s instructions (Qiagen). Purified eluate was used for raising antibodies in rabbits. For expression of JEV-C in mammalian cells, JEV-C was amplified from pET23b-JEV-C clone using the following forward and reverse primers containing Kpn I and Not I restriction sites: 5′-gtggtaccatgactaaaaaaccaggag-3′ and 5′-tagcggccgctcagtggtggtggtggtg-3′. The PCR product was cloned into pTNT vector (Promega) and from there to pEF1-myc-His-C as a Kpn I – Not I fragment so as to contain a c-terminal myc- and His-tag. All clones obtained were verified by sequencing. Caco-2 cells stably expressing JEV-C was generated by transfection of the pEF1-JEV-C-myc-His plasmid using Lipofectamine 2000 as per the manufacturer’s instructions (Invitrogen). Capsid expression was detected by both immunofluorescence and western blot analysis using mouse monoclonal anti-His antibodies (Clone His-H8, Abcam). Two clones with low and high expression of JEV-C were selected for all the experiments described.

### Virus Growth Curve

Approximately 4×10^4^ Caco-2 cells were seeded onto 6.5 mm diameter, 3 µm pore size polycarbonate membrane trans-wells (Costar) and fresh medium was added at 2 d intervals. Trans-epithelial electrical resistance (TER) of the monolayers was measured daily using a Millicell ERS-2 Volt-Ohm Meter (Millipore) and chopstick-type electrodes. Monolayer was allowed to attain maximum TER values before infection with JEV, which was usually around 5 or 6 days post-seeding. Virus (5 pfu/cell) containing medium was added onto the upper chamber of the trans-well inserts (100 µl) and medium without virus was added into the lower chamber (200 µl) and cells were incubated at 37°C on the rocker for 1 h. Virus inoculum was removed and cells were washed twice in phosphate buffered saline (PBS) or Hanks Balanced Salt Solution (HBSS), and re-fed with normal growth medium. Culture supernatants from upper and lower chambers were collected at indicated times and viral titers were determined by plaque assays on PS cells. Primary HUVEC was grown in 24-well plates and infected with an MOI of 5 pfu/cell as above and supernatants at the indicated time-points were collected for determining viral titers by plaque assay. Cell lysates were prepared from mock- or JEV-infected cells at 48 h p.i. as described below.

### Western Blot

Cells grown in 35 mm dishes were infected with JEV (MOI of 5 pfu/cell). Cell lysates were prepared at indicated times post-infection by washing cells twice with cold PBS on ice and cells were lysed by scraping cells into 200 µl of RIPA buffer (50 mM Tris-HCl pH 8.0, 150 mM NaCl, 1% IGEPAL CA-630, 0.5% sodium deoxycholate, 0.1% SDS with protease inhibitor mix (Roche) and 1 mM PMSF). Lysates were incubated on ice for 10 min and centrifuged at 12,000×g for 15 min at 4°C. Supernatants were boiled in 1X Laemmli buffer and resolved on a SDS-PAGE. Gels were transferred onto Immobilon-PVDF (Millipore) membranes for 2 h and analyzed by probing the blots with the indicated primary antibodies (rabbit anti-claudin-1 (Invitrogen), rabbit anti-occludin (Invitrogen), mouse anti-ZO1 (BD Biosciences), rabbit anti-β-catenin (Abcam), rabbit anti-α-catenin (Thermo Scientific), mouse anti-E-cadherin (Thermo Scientific-Pierce) goat anti RIG-I (Santa Cruz), rabbit anti-IRF-3 (Cell Signaling Technology), rabbit anti-MDA5 (Abcam), mouse anti-β-actin (Sigma-Aldrich). JEV infection was detected by a rabbit polyclonal antibody raised against the recombinant JEV capsid protein purified in bacteria as described above or by a commercial rabbit anti-JEV antibody (Abcam). Primary antibody incubation was followed by HRP-conjugated secondary antibodies (Invitrogen) and developed by enhanced chemiluminescence substrate (Thermo Scientific-Pierce). For the inhibitor experiments, cells were washed once at 23 h pi with HBSS and DMEM with 2% FBS and the indicated concentrations of following inhibitors were added on to the respective wells: 20 µM Z-VAD (OMe)-FMK (pan-caspase inhibitor), 50 µM MG-132, 0.5 mM N^G^-Nitro-L-arginine Methyl Ester (L-NMMA), 10 µM diphenyleneiodonium Chloride (DPI) and 200 nM bafilomycin A1. Cells were grown for a further 24 h in the presence of inhibitors and lysates were prepared as described above. Densitometry of the blots were performed using ImageJ software (NIH, USA).

### Immunofluorescence

Cells were infected as above and TER values were monitored post-infection. At 42–44 h pi when TER values of JEV-infected samples were about 40% of the control, cells were fixed in methanol and processed for immunofluorescence as described with some modifications (http://www.zonapse.net/protocols/id6.html). Culture inserts were washed twice with PBS and incubated with cold methanol at −20°C for 10 min or overnight followed by washing with PBS. Cells were further incubated with IMF buffer (20 mM HEPES, pH 7.5, 0.1% Triton-X-100, 150 mM sodium chloride, 5 mM EDTA and 0.02% sodium azide as a preservative) for 5 min at room temperature (RT) and all further washes were performed with IMF buffer. Non-specific antibody binding sites were blocked by incubating with IMF buffer containing 2% normal goat serum for 10 min at RT. Cells were washed three times followed by incubation with anti-E (pan flavivirus monoclonal-4G2 from Millipore) and other indicated antibodies for 1 h at RT followed by Alexa flour-conjugated secondary antibodies for 1 h at RT. Membranes were cut out using a scalpel blade, mounted on glass slides with Prolong anti-fade reagent with DAPI (Invitrogen) to stain the nuclei and covered with cover-slips and left to dry overnight in dark at RT. Images were acquired using Olympus FLUOVIEW FV1000 confocal system with 60× or 100× objective.

### Fluorescein Leakage Test

Leakage of fluorescein dye across the monolayer was measured as previously described by [19]. Briefly, cells were infected as above with JEV. At 42–44 h pi, cells were washed twice with HBSS. The top compartment was replaced with 0.2 ml of DMEM (without phenol red) containing 0.01% water-soluble fluorescein (Sigma-Aldrich) and 500 µl of medium without dye was added to the bottom compartment and placed in the incubator for one hour at 37°C. 100 µl duplicate aliquots were removed from the bottom compartment and amount of fluorescein dye was measured in a multi-mode reader (Biotek) at 480/530 nm (excitation/emission).

### Luminex Assays

Cells were infected as above and culture supernatants were collected at 42–44 h pi. 25 µl of the supernatant was used for assaying the cytokines and MMPs using antibody non-magnetic bead arrays as per the manufacturer’s instructions (Millipore). Absolute amount of analytes present in the medium was estimated by standard curve generated using known amount of analyte provided in the assay kit. Assay plates were read using Luminex-200 system and data was analyzed by xPONENT analysis software (Luminex Corporation).

### Quantitative Real Time PCR

Total cellular RNA was isolated from mock- or JEV-infected cells at the indicated time-points post-infection using Trizol reagent according to the manufacturer’s instructions (Invitrogen). 4 µg of RNA was treated with 4.0 unit of RNAse free DNAse I (MBI Fermentas) for 30 minutes at 37°C and then 10 minutes at 65°C to inactivate the DNAse. cDNA synthesis was performed using Omniscript Reverse Transcription System (Qiagen) using random hexamers as per manufacturer’s instructions. 100 ng of cDNA was used to determine JEV genome copy numbers using Taqman quantitative RT-PCR reagent (Life Technologies) using primers and probe described previously [20]. 200 ng of cDNA was used for detection of various genes using Fast SYBR Green quantitative RT-PCR reagent (Life Technologies) using primers as listed in Table S1. Reaction conditions used were as follows: (50°C- 2′; 95°C- 10′ followed by 95°C- 15′′; 60°C- 1′ for 40 cycles). GAPDH or actin transcripts levels were measured in parallel for normalization. Relative expression was calculated using comparative threshold cycle method.

### ROS Measurement

Cells were grown in 12-well plates and infected as above except that the growth medium without phenol red was used throughout to avoid background interference in the measurements. At indicated time points post-infection, cells were washed twice with HBSS and incubated with medium containing 1% FBS and 10 µM of carboxyl analog of 2′,7′-dichlorodihydrofluorescein diacetate (carboxy-H_2_DCFDA) for one hour at 37°C. Upon cleavage of the acetate groups by intracellular esterases and oxidation, the nonfluorescent H_2_DCFDA is converted to the highly fluorescent 2′,7′-dichlorofluorescein (DCF). Cells were detached and collected in HBSS and the amount of intracellular DCF was measured by FACS.

### Statistical Analysis

All statistical analyses were performed using Prism software (GraphPad Software, Inc.). P values were determined by unpaired, non-parametric, two-tailed t-tests. P value of less than 0.05 was considered as significant.

## Results

### Effect of JEV Infection on the Expression of Junctional Proteins

**Figure 1:**
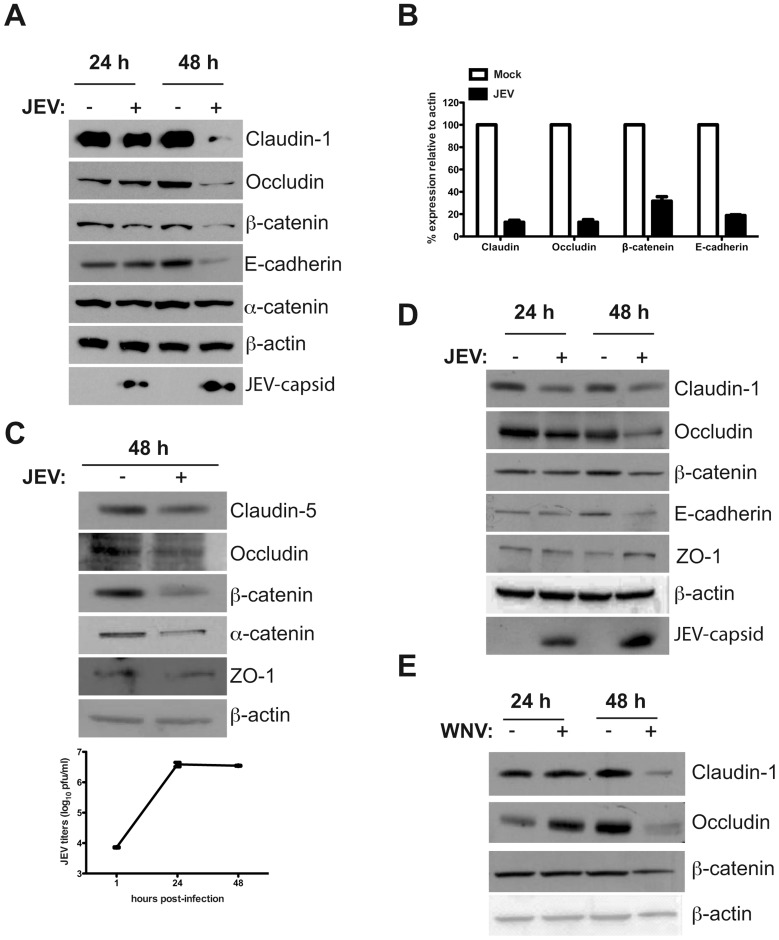
JEV infected cells have reduced expression of junctional proteins. (A) Caco-2 cells were infected with JEV as described in materials and methods and cell lysates were collected at indicated times post-infection and analyzed by western blot analysis for the indicated proteins. (B) Densitometry of western blots of two experiments performed with Caco-2 lysates 48 h p.i. Signal intensity is normalized to β-actin levels from the same blots. Error bars indicate mean with SD. (C) HUVEC cells were infected with JEV as described in materials and methods and cell lysates were collected at indicated times post-infection and analyzed by western blot analysis for the indicated proteins and viral titers determined by plaque assay at the indicated time post-infection is shown. (D) Huh7 cells were infected with JEV or WNV (E) as described in materials and methods and cell lysates were collected at indicated times post-infection and analyzed by western blot analysis for the indicated proteins.

We investigated the effect of JEV infection on junctional proteins (both tight and adherens junctions) in caco-2, Huh7 and primary HUVECs. We performed western blot analysis of total cell lysates prepared from mock- and JEV-infected cells for various TJ and AJ proteins at the indicated time post-infection. In caco-2 cells, the levels of TJ membrane proteins claudin-1 and occludin and also the adherens junction proteins β-catenin and E-cadherin was drastically reduced at day 2 post-infection whereas the effect was marginal on α-catenin (Figure 1A and 1B). We confirmed these observations in primary HUVEC, which was found to support JEV infection. As in the case of epithelial cells, we found that JEV-infected primary endothelial cells had lower levels of tight junction proteins claudin-5 (an endothelial specific claudin family member) and occludin and AJ proteins α and β-catenin whereas ZO-1 levels were only marginally reduced (Figure 1C). Similarly, in Huh7 cells, a reduction in both TJ (claudin-1 and occludin) and AJ (β-catenin and E-cadherin) proteins was observed however ZO-1 was unaffected (Figure 1D). We observed a similar effect on TJ and AJ proteins when Huh7 cells were infected with WNV (Figure 1E). These results suggest that JEV infection specifically affects a subset of TJ and AJ proteins in a variety of non-neuronal cell types.

### JEV Replication in Intestinal Epithelial Cells

**Figure 2:**
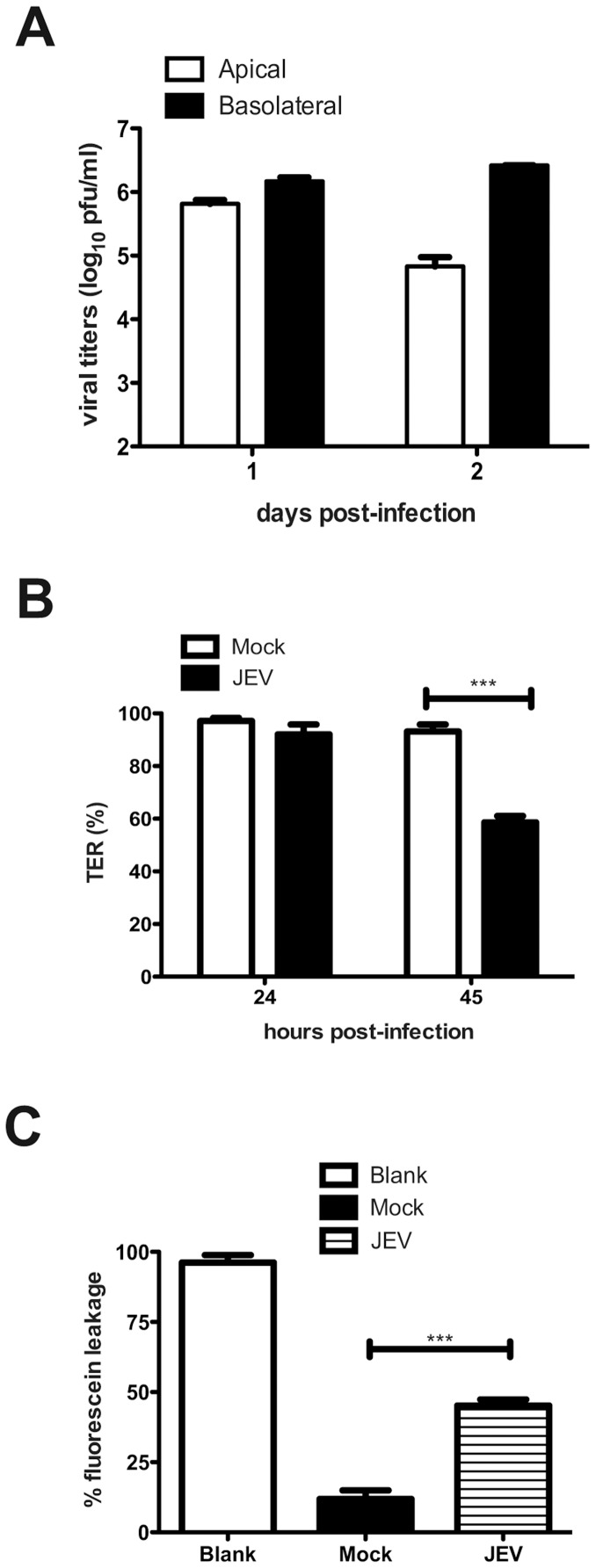
JEV replication and effects on permeability barrier in Caco-2 cells. (A) Cells grown on trans-well inserts were infected with JEV with an MOI of 5 pfu/cell and viral titers in the apical and basolateral media was measured by plaque assay at the indicated times post-infection. (B) TER was measured from cells grown on trans-wells and infected with JEV as above. (C) JEV infected cells were incubated with soluble fluorescein and the amount of fluorescein passing from apical to basolateral side was measured as described in materials and methods. The figures are representative of three or more experiments performed with three or more replicates. Error bars indicate mean ± s.d. *** p<0.0001 as determined by two-tailed t-test.

The reduction in tight and adherens junction proteins by JEV infection in epithelial and endothelial cells may possibly lead to disruption of permeability barrier functions of these cells. Both epithelial and endothelial cells form tight junctions, however, epithelial cells have been shown to generate a strong permeability barrier as compared to endothelial cells in cell culture [21], [22]. Caco-2 cells have been used extensively for studying polarized epithelial cell functions. Therefore, we further tested the effect of JEV infection on epithelial permeability barrier functions in these cells. Cells were grown on trans-well inserts and infected with JEV from the apical surface. Viral titers were measured from both apical and basolateral medium on day 1 and 2 post-infection. In contrast to our previous report on WNV [23], JEV titers were more than 10-fold higher in the medium from the basolateral surface as compared to the apical surface suggesting that either JEV exits these cells preferentially via basolateral route or the virus was capable of breaching the permeability barrier and reach the basolateral medium from apical side (Figure 2A). To differentiate between these two possibilities, we next examined if JEV infection causes a compromise in barrier functions of these cells by measuring the TER across the monolayer as a measure of barrier integrity in infected cells. We observed close to 50% reduction in TER values of JEV-infected cells at 45 h p.i. compared to mock-infection indicating an effect on barrier functions due to JEV infection (Figure 2B). To further assess the intactness of the barrier functionally, we incubated JEV-infected cells with water-soluble fluorescein dye by adding the dye on the apical side and measured the amount of dye that reached the basolateral side, which indicates the leakiness of tight junctions that maintain permeability barrier in these cells. As shown in Figure 2C, we observed a 40% increase in the amount of dye in basolateral medium in JEV-infected cells compared to mock-infection further demonstrating a compromise in barrier functions in JEV-infected cells. We tested the viability of cells at this time-point by trypan blue exclusion and found no difference between mock and JEV-infected cells (Figure S1A). JEV infection was not cytotoxic at this time-point as measured by lactate dehydrogenase release assay suggesting that the observed effect on barrier functions is not due to cell death or cytotoxicity (Figure S1B).

### Inhibition of JEV Replication Blocks Epithelial Barrier Disruption

**Figure 3:**
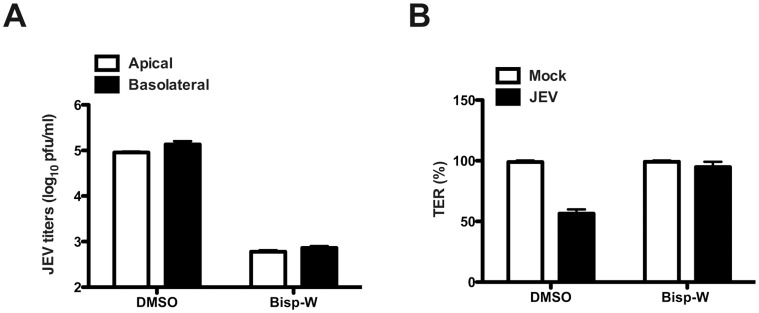
Inhibition of JEV replication blocks permeability barrier disruption. (A) Cells grown on trans-well inserts were infected with JEV with an MOI of 5 pfu/cell and treated with 20 µM Bisp-W at 1 h post-infection. Viral titer in the apical and basolateral media was measured by plaque assay after two days post-infection. (B) TER was measured from cells grown on trans-wells and infected and treated as above.

We have recently identified amino acid conjugates of 3,7-diazabicyclo[3.3.1]nonane, commonly called bispidine (Bisp-W), as novel inhibitor of JEV replication [18]. To identify if JEV replication is required for TER disruption, caco-2 cells were infected with JEV and Bisp-W was added at 1 h p.i. to the growth medium and cultured for 48 h. TER was monitored as before and viral titers from apical and basolateral medium was estimated by plaque assays. As expected, Bisp-W inhibited virus production by over 100-fold and block in virus replication prevented TER disruption demonstrating that virus replication is essential for barrier disruption (Figure 3A and 3B). Similarly, UV-inactivated virus was inefficient in disrupting TER suggesting that active viral replication is required for barrier disruption (data not shown).

### Localization of Junctional Proteins in JEV Infection

**Figure 4:**
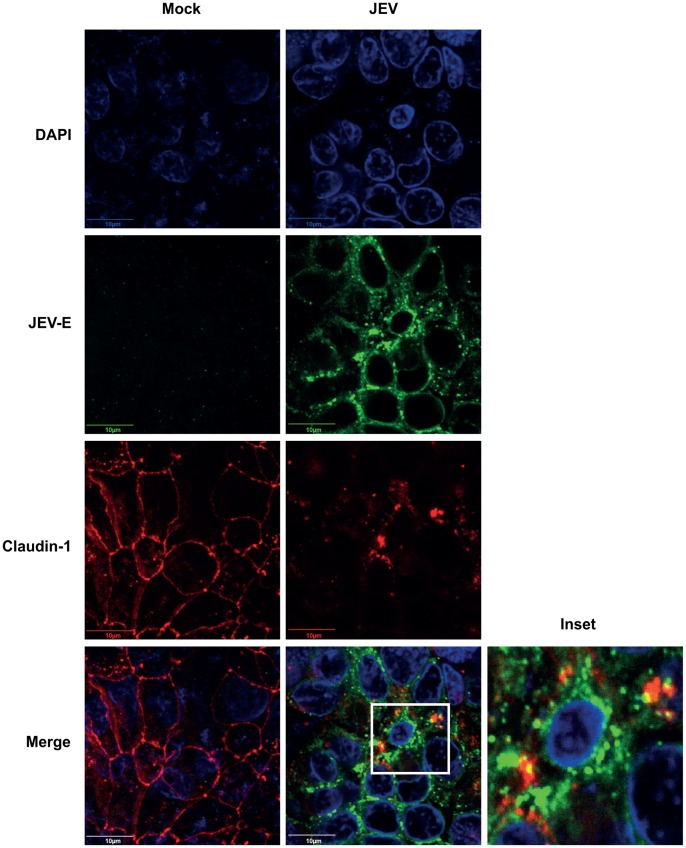
JEV infection alters the localization of claudin-1. Caco-2 cells grown on trans-wells were infected with JEV and at 42 h p.i. cells were fixed and stained for JEV and claudin-1 as described in materials and methods. Marked area in the merge image is shown separately to demonstrate the colocalization of claudin-1 and JEV-E.

We next investigated if JEV mediated disruption of epithelial barrier function is due to mislocalization of TJ components. To determine the localization of various TJ and AJ proteins in JEV-infected cells, we performed immunofluorescence analysis of mock-and JEV-infected cells at the time when TER values in infected cells were down to about 50% of mock-infected cells. We observed a drastic effect on the quantity and localization of claudin-1 in JEV-infected cells (Figure 4). Claudin-1 in infected cells was barely localized to TJs and redistributed to intracellular compartments partially colocalizing with the virus (Figure 4). In contrast, the localization of β-catenin was only marginally perturbed in JEV-infected cells (Figure S2A) and the localization of occludin and ZO-1 was unaltered upon infection although the signal intensity of these two proteins was lower in infected cells as compared to mock infection (Figure S2B and S2C) reconfirming the western blot results. Altogether, these data suggest that JEV may target some of the junctional proteins for degradation and thereby causing disruption of epithelial barrier functions.

### JEV Infection Induces Antiviral and Stress Response Genes in Epithelial Cells

**Figure 5:**
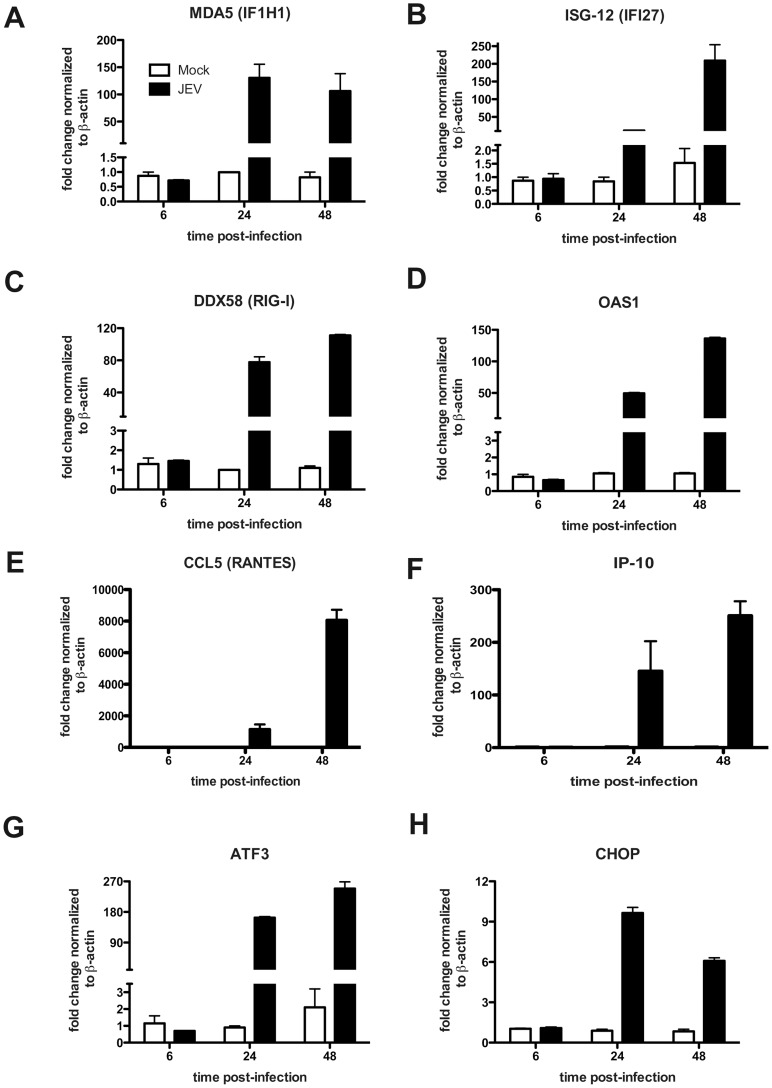
JEV infection induces antiviral and cellular stress response genes. Caco-2 cells were infected with JEV and total RNA was prepared from cells at indicated times post-infection. mRNA levels of indicated genes was quantitated by real time PCR as described in materials and methods. GAPDH or β-actin mRNA levels were quantitated in parallel for normalization. The figures are representative of two experiments performed with three replicates. Error bars indicate mean ± s.d.

**Figure 6:**
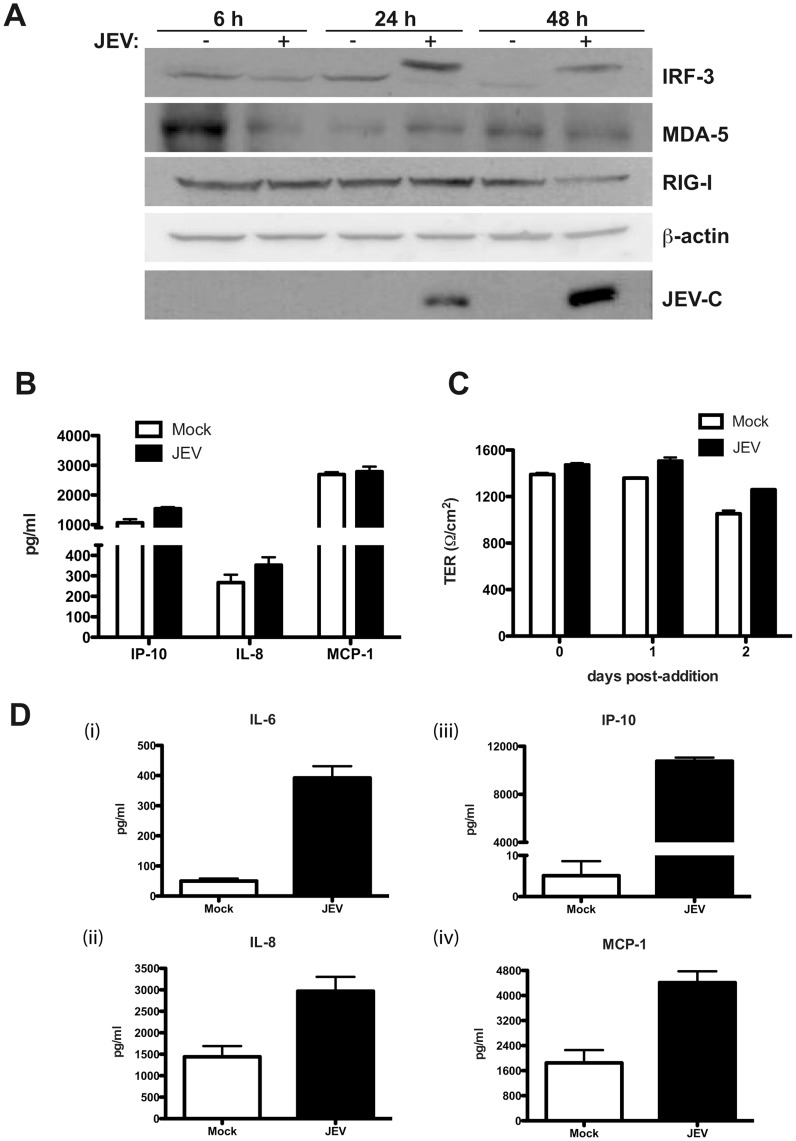
JEV mediated disruption of tight junctions is independent of secreted factors. (A) Caco-2 cells were infected with JEV and cells were collected at indicated time points for preparation of cell lysates. Induction/activation of the indicated proteins was detected by western blot analysis. β-actin serves as loading control and JEV infection is indicated by the expression of capsid protein (JEV-C). (B) The amount of indicated cytokines in infected culture supernatants were measured by Luminex bead assays as described in materials and methods. The figures are representative of three experiments performed with two or more replicates. Error bars indicate mean ± s.d. (C) Clarified supernatants from JEV-infected cells were added onto naïve caco-2 cells grown on trans-wells and TER levels were monitored for indicated periods. The figures are representative of three experiments performed with two or more replicates. Error bars indicate mean ± s.d. (D) The amount of indicated cytokines in infected culture supernatants of HUVECs were measured by Luminex bead assays at 48 h pi as described in materials and methods. Error bars indicate mean with SEM of three replicates.

A number of earlier studies have reported induction of inflammatory response in JEV-infected microglial and neuronal cells as a causative factor leading to neuronal death [24]–[27]. We investigated if infection of epithelial cells by JEV induces a similar antiviral and cellular stress response. We measured the transcripts of RNA helicases involved in antiviral responses namely interferon-induced with helicase C domain 1/melanoma differentiation associated gene 5 (IFIH1/MDA5) and retinoic acid-inducible gene-I/DEAD box polypeptide 58 (RIG-I/DDX58) [28] and interferon-stimulated genes ISG-12/IFI27 and 2′-5′-oligoadenylate synthetase 1 (OAS1) and the chemokine CCL5/RANTES and cytokine IP-10. We observed a robust induction of all the above genes by 24 h pi which was sustained till 48 h pi indicating the activation of antiviral pathways in these cells upon JEV infection (Figure 5A–F). We next tested for activation of cellular stress response pathways in response to JEV infection by using activating transcription factor 3 (ATF3) and CCAAT/enhancer-binding protein homologous protein (CHOP) transcripts as markers. In agreement with previous reports, we found induction of these genes by 24 h p.i. which was sustained even at 48 h p.i., although, CHOP induction was reduced at 48 h p.i. relative to 24 h p.i. suggesting that the virus may subvert the prolonged activation of stress response genes to avoid cell death (Figure 5G–H). To further confirm the induction of antiviral state in these cells we analyzed the activation of interferon regulatory factor 3 (IRF3) upon JEV infection by western blot analysis. We observed a high-molecular weight band of IRF3, which presumably represents the phosphorylated form of IRF3, by 24 h p.i. and 48 h p.i. indicating the induction of antiviral response which is in confirmation with earlier report on WNV-infected epithelial cells [29]. However, in contrast to the qPCR data, we did not observe induction of MDA5 and/or RIG-I at the protein levels indicating that JEV infection possibly blocks induction of these proteins post-transcriptionally and IRF3 activation is independent of MDA5 or RIG-I in these cells (Figure 6A). To further measure the induction of inflammatory response, we used multiplex bead assays to detect the cytokines and MMPs secreted in the infected culture supernatants. The levels of TNF-α, IFN-α, IFN-γ, IL-6 and IL-12 were below the limit of detection of the assays. Only IP-10, IL-8 and MCP-1 were detectable in the culture supernatants and JEV infection did not lead to induction of these inflammatory cytokines (Figure 6B). Similarly, we were able to detect MMP-1, 7 and 10 in both mock and JEV-infected supernatant with no effect on the levels of these MMPs upon JEV infection (Figure S3). The amount of MMP-2 and MMP-9 was below the level of detection. These results suggest that despite the induction of a strong antiviral response at transcriptional level, JEV infection in these cells does not lead to increased production of inflammatory cytokines or matrix metalloproteinases which have been implicated in tight junction disruption in other cell types upon flavivirus infection [30]–[33]. To further confirm if disruption of epithelial barrier functions is due to any other secreted factors, we treated naïve caco-2 cells with infected cell culture supernatants from cells collected 44 h p.i. (clarified to remove virus) and observed for reduction in TER for the indicated time points. JEV-infected culture supernatants were not able to disrupt barrier functions even at 48 h post-addition of the infected culture supernatants (Figure 6C). These data suggest that the disruption of barrier functions observed is due to the effect of JEV replication somehow affecting the TJ and AJ components and not due to secreted factors from infected cells.

We showed that HUVECs support JEV infection, which affected some of the junctional proteins. We next determined the cytokine profile in supernatants from JEV-infected HUVECs and in contrast to caco-2 cells, we were able to detect induction of IL-6 (8-fold), IL-8 (2-fold), IP-10 (2000-fold) and MCP-1 (about 2.5 fold) (Figure 6D). However, TNF-α, IFN-α, IFN-γ and IL-12 were below the limit of detection of the assays. A previous report on JEV-infected endothelial cells isolated from rats also failed to detect TNF-α indicating that the high levels of TNF-α observed in the brains of JEV-infected mice is contributed by non-endothelial cells such as microglial cells [26], [34]. This data suggests that while epithelial cells support JEV infection by suppressing innate immune responses, infection of endothelial cells with JEV induces robust inflammatory response. It is plausible that the secretion of some of these cytokines/chemokines by endothelial cells may contribute to JEV pathogenesis *in vivo*.

### Claudin-1 is Targeted to Lysosomal Degradation in JEV-infected Cells

**Figure 7:**
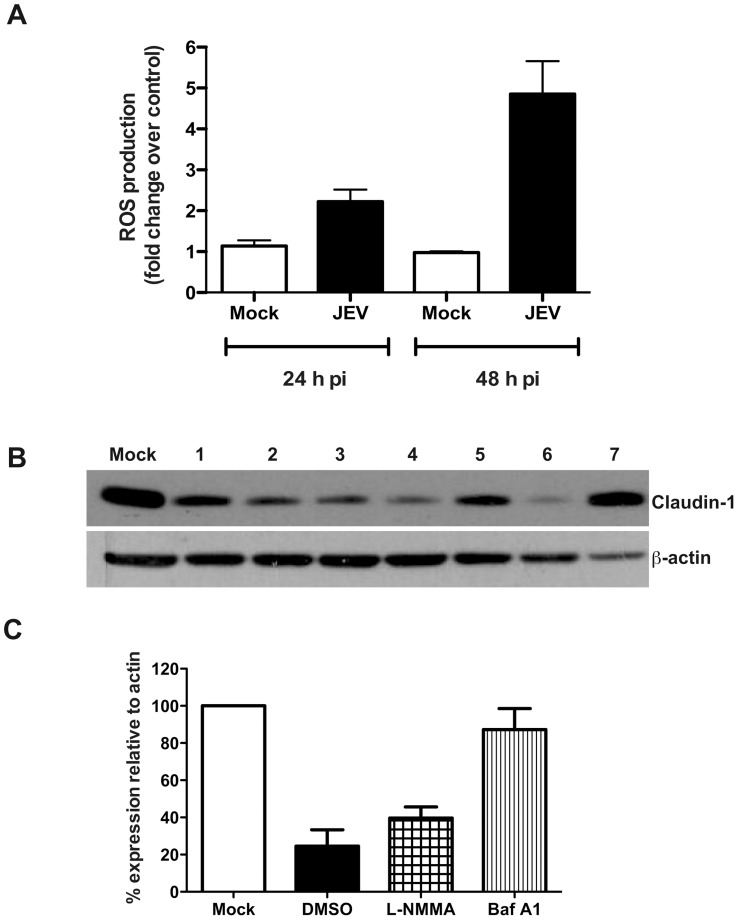
Effect of inhibitors on claudin-1 degradation. (A) Caco-2 cells were infected with JEV and at the indicated times post-infection, cells were incubated with carboxy-H_2_DCFDA and the amount of fluorescent 2′,7′-dichlorofluorescein produced was detected by FACS as a measure of ROS production as described in materials and methods. (B) Cells were mock- or JEV-infected and at 23 h p.i. incubated in growth medium containing: 1) Untreated 2) DMSO (equal volume) 3) 20 µM Z-VAD (OMe)-FMK (4) 50 µM MG-132 (5) 0.5 mM L-NMMA (6) 10 µM DPI (7) 200 nM bafilomycin A1. Cell lysates were prepared 24 h post-addition of the compounds and the levels of claudin-1 was detected by western blot analysis. β-actin was also detected as a control for equal loading. (C) Densitometry of western blots of two experiments performed with lysates prepared from caco-2 cells mock-infected or infected with JEV and treated with DMSO or L-NMMA or bafilomycin A1. Signal intensity is normalized to actin levels from the same blots. Error bars indicate mean with SEM.

JEV infection has been shown to induce generation of reactive oxygen species (ROS) *in vivo* in mouse models [35]. Oxidative stress is known to cause perturbation in permeability barrier functions in epithelial and endothelial cells [36]–[39]. Therefore, we next measured the ROS levels in Caco-2 cells upon JEV infection. We observed a two-fold increase in the ROS levels in JEV-infected cells at 24 h p.i. which increased to about five-fold as compared to mock infection at 48 h p.i. demonstrating the induction of oxidative stress in these cells due to JEV infection (Figure 7A). Previous reports, including ours, have shown that a subset of TJ proteins are targeted to lysosomes for degradation in epithelial and endothelial cells infected with WNV [23], [40]. Therefore, to identify the pathway involved in tight junction disruption upon JEV infection, we used a number of inhibitors to rescue claudin-1 from degradation. Caco-2 cells were infected with JEV and at around 23 h p.i. inhibitors were added on to the cells and incubated for a further 24 hours. Cell lysates were prepared and claudin-1 levels were detected by western blot analysis. We found that the pan-caspase inhibitor (Z-VAD-OMe-FMK), proteasomal inhibitor (MG-132) and inhibitor of oxidative stress (DPI) failed to prevent claudin-1 degradation, however, cells treated with nitric oxide synthase (L-NMMA) partially rescued claudin-1 degradation. The vacuolar ATPase proton pump inhibitor bafilomycin A1 blocked claudin-1 degradation and claudin-1 levels in bafilomycin A1-treated cells were almost similar to mock-infected cells (Figure 7B and 7C) suggesting that, as in the case of WNV, claudin-1 is targeted for lysosomal degradation in JEV-infected cells which possibly leads to dysfunction in barrier properties.

### Cells Expressing JEV-capsid Alone Display Compromised Barrier Functions

**Figure 8:**
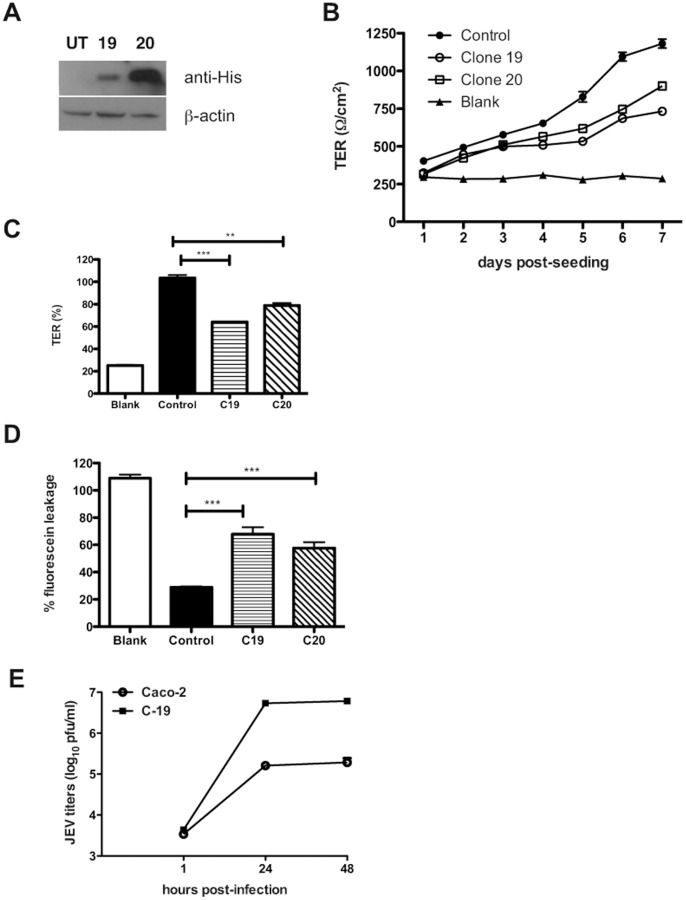
JEV capsid alone alters permeability barrier functions. (A) Lysates were prepared from two caco-2 clones expressing JEV-C and analyzed by western blot for expression of JEV-C (anti-His antibody) or β-actin. (B) TER was measured in caco-2 capsid clones grown on trans-wells as described above. (C) **%** TER levels in the indicated cells at day 7 post-seeding is shown. (D) Control or JEV-C caco-2 clones were incubated with soluble fluorescein and the amount of fluorescein passing from apical to basolateral side was measured as described in materials and methods. (E) Caco-2 and capsid clone-19 cells were infected with JEV (5 pfu/cell) and supernatants were collected at indicated time post-infection. Viral titer in the supernatant was measured by plaque assay. The figures are representative of two experiments performed with three replicates. Error bars indicate mean ± s.d. *** p<0.0001 and ** p<0.002 as determined by two-tailed t-test.

**Figure 9:**
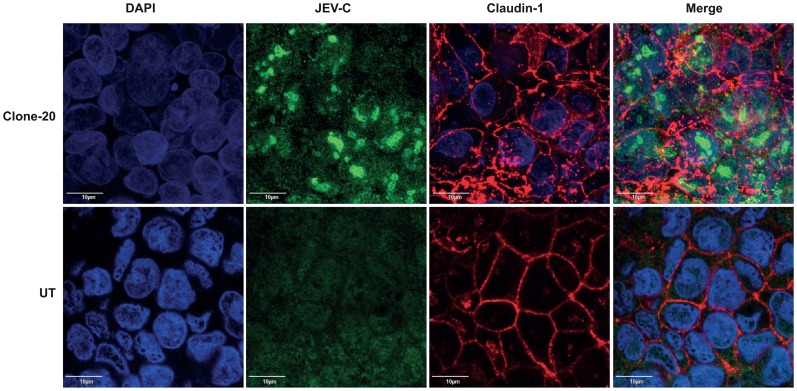
JEV-C expression alters the localization of claudin-1. Caco-2-C cells grown on trans-wells were fixed and stained for JEV-C (anti-His-Green) and claudin-1 (Red) as described in materials and methods.

Our studies with WNV had identified a role for WNV structural proteins in TJ disruption and WNV-capsid alone was capable of affecting the permeability barrier functions [23]. To further test if JEV-capsid has a similar function, we generated stable Caco-2 clones expressing recombinant JEV-capsid. We selected two cell lines with high and low expression of JEV-C as determined by western blot analysis for further studies (Figure 8A). We measured the TER values of cells up to 7 days post-seeding, the time-point when untransfected cells attain maximum TER. We found that although there was an increase in the TER values in both the clones over time, the values attained at day 7 by both the JEV-C clones were about 40% lower as compared to control cells (Figure 8B and 8C). We next tested the leakiness off the barrier in these cells by fluorescein-leakage test and found that these clones allowed two-fold more amount of dye to pass through as compared to the control cells suggesting that the epithelial barrier function is partially perturbed in cells expressing JEV-capsid (Figure 8D). We also assessed if leaky junctions in Caco-2-capsid clones affects JEV infection either positively or negatively by infecting these cells with JEV and viral titers from the supernatants were measured at indicated time points. We observed a 10-fold increase in viral titers in cells expressing JEV-C suggesting that compromising the epithelial barrier functions may play an important role in JEV infectious virus production (Figure 8E). We next performed immunofluorescence analysis to observe the localization of claudin-1 and β-catenin in one of the capsid clones. JEV-C expression was found to localize to the nucleus as distinct circular and dumbbell shaped structures, which is similar to published reports on flaviviral capsids [41]–[45]. Surprisingly, capsid expression led to altered localization pattern of claudin-1 although the levels of claudin-1 and other junctional proteins in these cells was not significantly reduced suggesting that capsid expression may have a negative effect on formation or maintenance of tight junctions (Figure S4 and Figure 9). Localization of β-catenin remained unperturbed in JEV-C clone suggesting that JEV-C expression specifically affects tight junction functions and may not impact the adherens junctions indicating a role for other JEV proteins in the overall effect on permeability barrier functions (Figure S5).

## Discussion

In this study we show that infection of epithelial and primary endothelial cells and hepatocytes with JEV affects the stability of a sub-set of TJ and AJ proteins. We propose that the effect of JEV infection on junctional proteins leads to disruption of cellular junctions resulting in perturbation of paracellular barrier functions of polarized cells. Using a novel inhibitor of JEV, we show that disruption of barrier functions requires JEV replication. JEV appears to specifically affect the TJ proteins from claudin family although the levels of other TJ components such as occludin and ZO-1 and also the adherens junction proteins E-cadherin, α- and β-catenin were lower in infected cells compared to uninfected cells suggesting a general effect of JEV on cellular junctions. However, localization studies showed a marked redistribution of claudin-1, but not occludin, ZO-1 or β-catenin, from junctions to intracellular compartments. The residual claudin-1 in infected cells colocalized with the virus further suggesting that JEV may directly affect claudin-1 localization and stability. We found that JEV infection of Caco-2 cells induced upregulation of genes involved in cellular stress response and antiviral response at the transcriptional level including the RNA helicases RIG-I and MDA5 and IRF-3. In an earlier report, RIG-I levels were found to be elevated in JEV-infected mouse brain tissues [27]. In our study, JEV-infection of epithelial cells *in vitro* failed to induce either RIG-I or MDA5, the two RNA helicases which have been shown act as RNA sensors for a variety of viruses [28], [46] suggesting cell type specific role for these innate immune response genes. In addition, our results also suggest that the activation of IRF-3 in these cells is independent of RIG-I which is in contrast with the previous report where expression of dominant negative forms of RIG-I ablated IRF3 activation in JEV-infected Vero and A549 cells [47]. However, despite the activation of IRF-3 and other interferon response genes, we did not detect any induction of interferons or other pro-inflammatory markers such as IP-10, IL-8 and MCP-1 in infected epithelial cells whereas JEV infection of HUVECs induced a strong inflammatory response leading to secretion of some of the pro-inflammatory cytokines/chemokines. Our results indicate that in Caco-2 cells disruption of cellular junctions by JEV is independent of secreted factors and, possibly, is a direct effect of infection. Our earlier study with WNV, a virus closely related to JEV, also showed a similar direct effect of WNV on TJ protein claudin-1 which was confirmed by Xu *et al* who demonstrated that WNV infection induces endocytosis and lysosomal degradation of a subset of TJ proteins [23], [40]. We had earlier reported that stable expression of WNV-capsid protein alone caused significant amount of barrier disruption and claudin-1 degradation in two clones expressing varied level of WNV-C. However, Xu *et al* monitored TJ protein levels in cells expressing WNV-C, using a lentiviral expression system, and failed to observe a similar effect. It should be noted that in the study by Xu *et al* TER levels were not monitored in capsid-expressing cells, which is a functional read-out for TJ integrity. It is likely that capsid expression levels using different approaches (stable transfection vs lentiviral vectors) could be the cause of this discrepancy. Our attempts to express JEV structural proteins (C-prM-E) in Caco-2 cells were not successful, however, we were able to generate cell lines expressing JEV-C alone. We show that cells expressing JEV-C had significantly compromised permeability barrier functions and altered claudin-1 distribution suggesting that JEV-C may play a role in the overall effect of JEV on TJ functions. In contrast to WNV-C expression in Caco-2 cells, we have not observed a marked difference in the protein levels of claudin-1 in JEV capsid-expressing cells. However, as TJ is a multi-protein complex, further investigations are necessary to ascertain the stability of other proteins of the TJ and AJ complex. In addition, since TJ is a dynamic structure, JEV-C may also perturb barrier functions by affecting the dynamics of TJ assembly and disassembly. The exact mechanism behind compromised barrier functions in JEV-C expressing cells is a matter for future investigation. These results further suggest that the effect of JEV on permeability barrier involves additional viral proteins.

TJ *per se* are not “tight” but are rather composed of size-selective pores which are determined by the homotypic and heterotypic interaction of the members of the claudin family. Thus claudins play a very important role in maintaining tissue homeostasis by regulating the paracellular transport which is confirmed by the observations that claudin-1 and claudin-5 knock-out mice die shortly after birth whereas occludin knock-out mice are viable with intact TJ albeit with other complex phenotypes [48]–[51]. TJ components have been shown to play a crucial role in the life-cycle of many viruses. Proteins of TJ complex promote viral infection by acting as receptors/co-receptors for viral entry [52], [53]. RNA viruses such as vesicular stomatitis virus, influenza virus and rotavirus have been shown to influence the permeability barrier functions of epithelial cells with or without cytopathic effects at later stages of infection [54], [55]. Kidney biopsies from patients infected with highly pathogenic hantaviruses revealed altered expression and localization of ZO-1 which correlated with disease severity [56]. In many cases, viral proteins responsible for affecting TJ functions have been identified. HIV-1 Tat protein alone was capable of perturbing TJs in primary human brain microvascular endothelial cells by down-regulating occludin mRNA and protein levels [57]. A number of previous reports have provided inconsistent and contradictory evidence on the effect of flavivirus infection on endothelial barriers. Liu *et al* reported that dengue virus infection of HUVECs had no effect on the permeability barrier and DENV was capable of reversing the effect of permeability disruption induced by recombinant TNF-α at earlier stages of infection but augment the same at later stages [58]. Although secreted factors from supernatants of dengue-infected monocytes or dendritic cells such as MCP-1 and MMP-9 were shown to mediate disruption of permeability functions in HUVECs these studies did not investigate the effect of direct infection of primary endothelial cells on barrier functions [30]–[33]. We demonstrate that in JEV-infected intestinal epithelial cells, tight junction disruption is a direct effect of JEV replication and is independent of secreted cytokines or MMPs. We further demonstrate that the capsid protein of JEV is capable of affecting the permeability barrier of epithelial cells and may play a significant role in viral dissemination as cells expressing recombinant JEV capsid protein produced more virus upon infection with JEV. Previous studies have reported a nuclear staining of flaviviral capsid proteins [23], [41]–[43], [45], [59], [60]. Infection with recombinant JEV harboring a mutation in the putative nuclear localization signal in the capsid protein led to production of increased number of defective particles compared to wild type virus and exhibited defects in viral RNA replication and translation in Vero cells. In addition, this virus was compromised in neuro-invasiveness thus demonstrating that the nuclear localization of JEV-C has a relevant function in JEV pathogenesis [44]. It would be interesting to determine if, in addition to binding viral RNA, flaviviral capsids in the nucleus regulate the gene expression of host proteins at the transcriptional level.

There are very few cases where the pathway or the mechanism of permeability barrier disruption has been characterized. Some examples include the involvement of oxidative stress in rhinovirus-mediated dissociation of ZO-1 from TJ complex [61]. Coxsackievirus B infection was shown to induce necrotic cell death as a result of cleavage of some of the junctional proteins by calpain, a calcium-dependent protease [62]. Our inhibitor data suggests that in JEV-infected epithelial cells, inhibition of apoptosis, proteasomal degradation or oxidative stress pathways failed to prevent claudin-1 degradation but bafilomycin A1, which prevents acidification of endosomes/lysosomes, was successful in blocking claudin-1 degradation indicating a role for lysosomal targeting of claudin-1 in barrier disruption. Previous report investigating neuro-invasion of WNV has clearly shown the reduction of both tight and adherens junction proteins and elevated levels of MMPs in the mouse brains only after viral entry into the CNS suggesting that the disruption of blood-brain barrier is a consequence of virus replication in the CNS [63]. Unlike WNV, there are no good animal models to study JEV pathogenesis in peripheral tissues and therefore, there is very little understanding of the progression of JEV infection from a mosquito bite to the CNS disease. Interestingly, a recent study investigating the effect of JEV on blood-brain-barrier integrity in a mouse model reported reduced expression of some of the tight junction genes (ZO-1, occludin, claudin-1 and claudin-5) and enhanced expression of adhesion molecules (ICAM1 and JAM) in the brains of JEV-infected *Stat1^−/−^* mice indicating that endothelial barriers may play a role in JEV neuro-invasion [64]. In contrast, another recent study showed that rat brain microvascular endothelial cells infected with JEV showed no effect on endothelial barriers [34]. Thus the actual role of endothelial barrier disruption in neuro-invasion warrants further investigation. Our future efforts will focus on identifying additional JEV proteins involved in perturbing tight junctions and understand the relevance of epithelial and endothelial barriers in JEV pathogenesis both in peripheral tissues and in the CNS in mouse models.

### Supporting Information

Supplementary figures and supplementary methods for this study are available as supporting information.

## Supporting Information

Figure S1 Cell viability and cytotoxicity of caco-2 cells infected with JEV. (A) Caco-2 cells grown in 12-well plates were infected with and MOI of 5 pfu/cell of JEV and cell viability at the indicated periods were measured by trypan blue exclusion. (B) Cytotoxicity was assessed by measuring the activity of lactate dehydrogenase in the supernatants. For lysis controls, detergent was added and the LDH activity in detergent treated sample was considered as 100% LDH release for comparison. Error bars indicate mean ± s.d.
(TIF)

Figure S2 Localization of occludin and ZO-1 in JEV infected caco-2 cells. Caco-2 cells grown on trans-wells were infected with JEV and at 42 h p.i. cells were fixed and stained for JEV and β-catenin (A) occludin (B) or ZO-1 (C) as described in materials and methods.
(PDF)

Figure S3 MMP levels in JEV-infected caco-2 supernatants. The amount of indicated MMPs in infected culture supernatants was measured by Luminex bead assays as described in materials and methods. The figures are representative of two experiments performed with two or more replicates. Error bars indicate mean with SEM.
(TIF)

Figure S4 Levels of TJ proteins in Caco-2 clones expressing JEV-C. Cell lysates from untransfected (UT) or three stable clones (5, 19, and 20) expressing JEV-C were analyzed by western blot analysis for the indicated junctional proteins.
(TIF)

Figure S5 JEV-C expression does not affect β-catenin localization. Caco-2-C cells grown on trans-wells were fixed and stained for JEV-C (anti-His) and β-catenin as described in materials and methods.
(TIF)

Table S1 Sequence of forward and reverse primer (5′-3′) used in qRT-PCR in this study.
(DOCX)

Methods S1 (DOCX)
