# Supplementary figures and images for: Japanese Encephalitis Virus Disrupts Cell-Cell Junctions and Affects the Epithelial Permeability Barrier Functions
Source: PLoS One. 2013 Jul 24;8(7):e69465. doi: 10.1371/journal.pone.0069465 (PMC3722119; doi:10.1371/journal.pone.0069465)

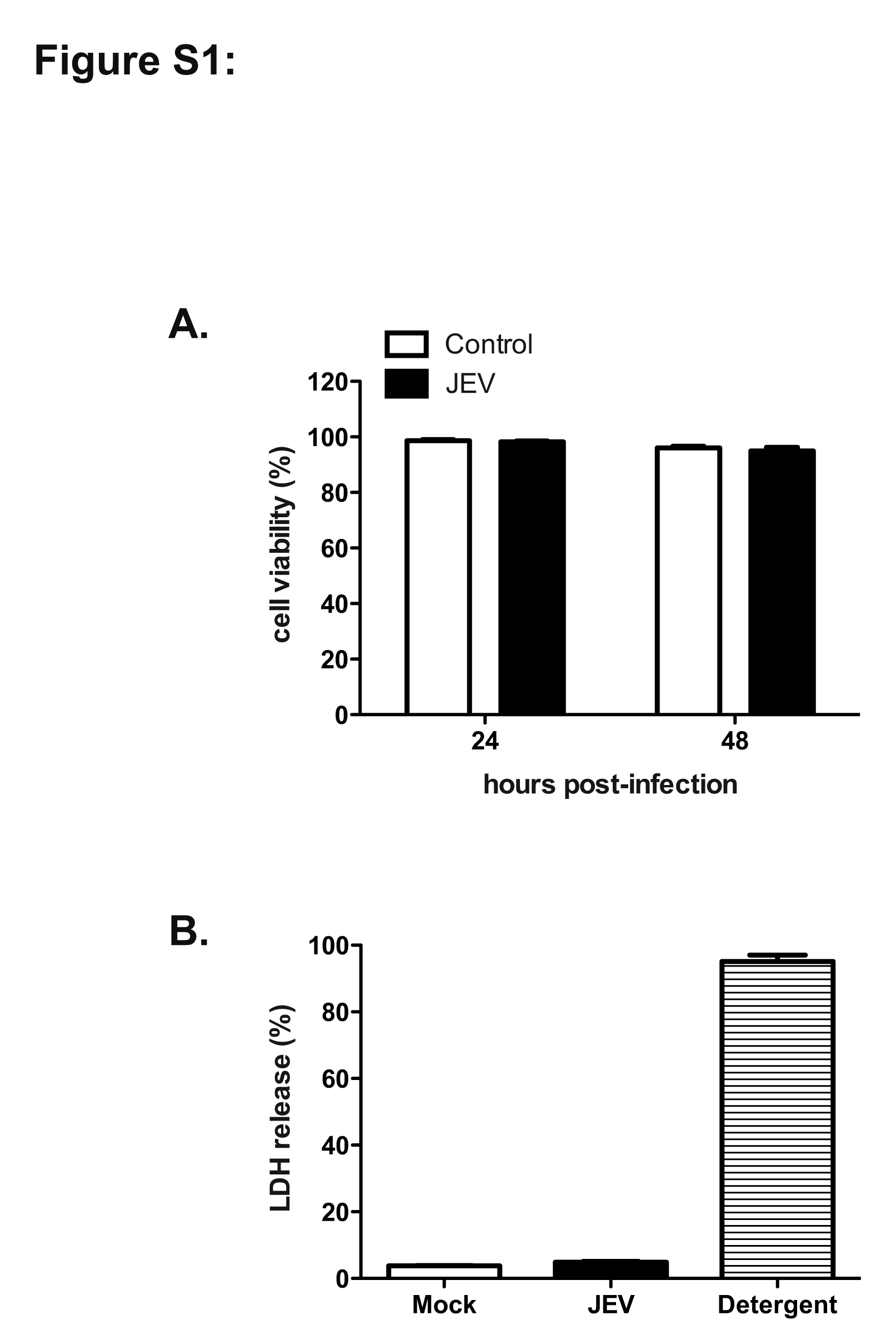

Supplement: Figure S1 — Cell viability and cytotoxicity of caco-2 cells infected with JEV. (A) Caco-2 cells grown in 12-well plates were infected with and MOI of 5 pfu/cell of JEV and cell viability at the indicated periods were measured by trypan blue exclusion. (B) Cytotoxicity was assessed by measuring the activity of lactate dehydrogenase in the supernatants. For lysis controls, detergent was added and the LDH activity in detergent treated sample was considered as 100% LDH release for comparison. Error bars indicate mean ± s.d. (TIF) [file pone.0069465.s001.tif]

**Figure S2A:**

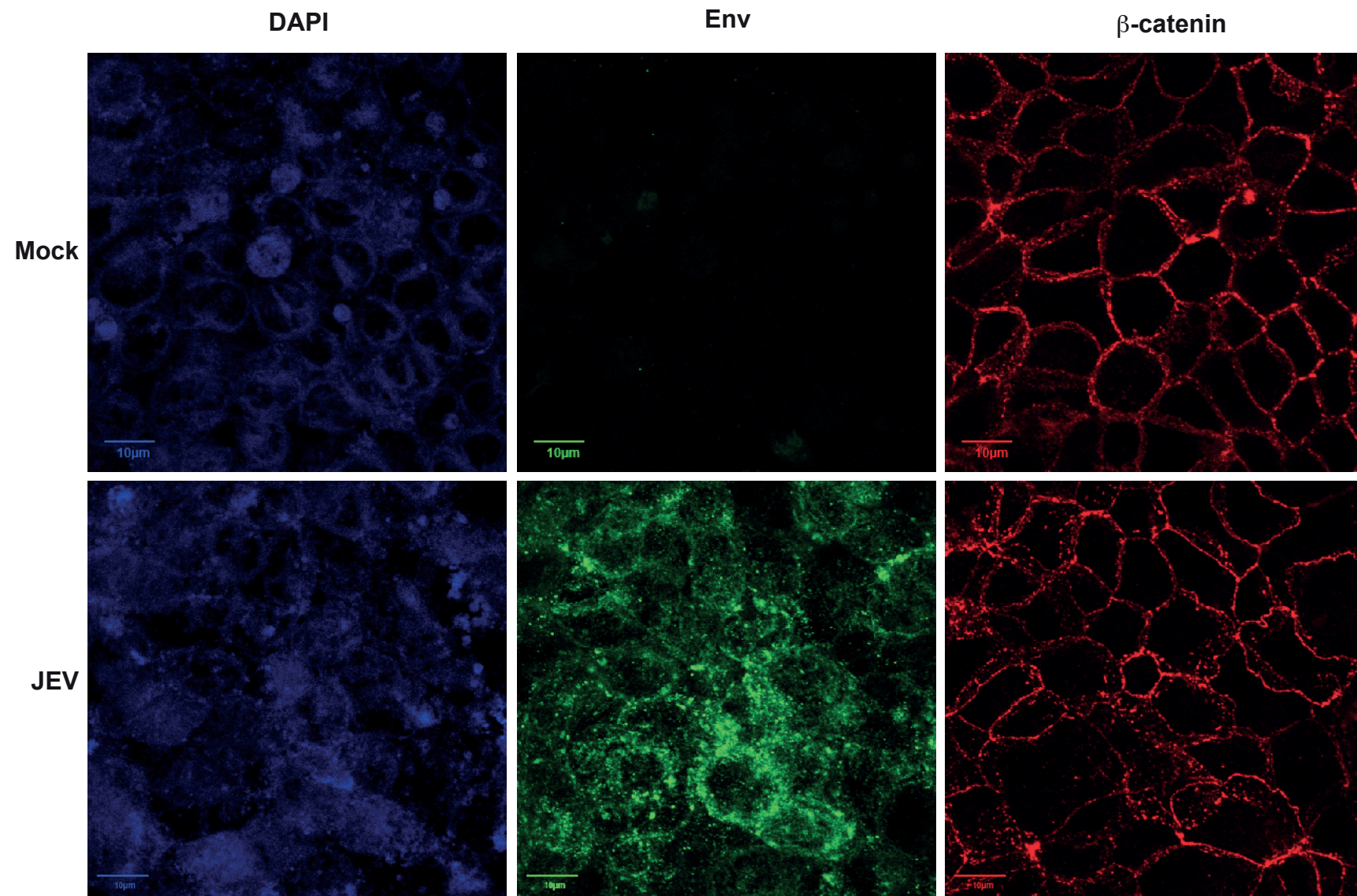

Figure S2B:

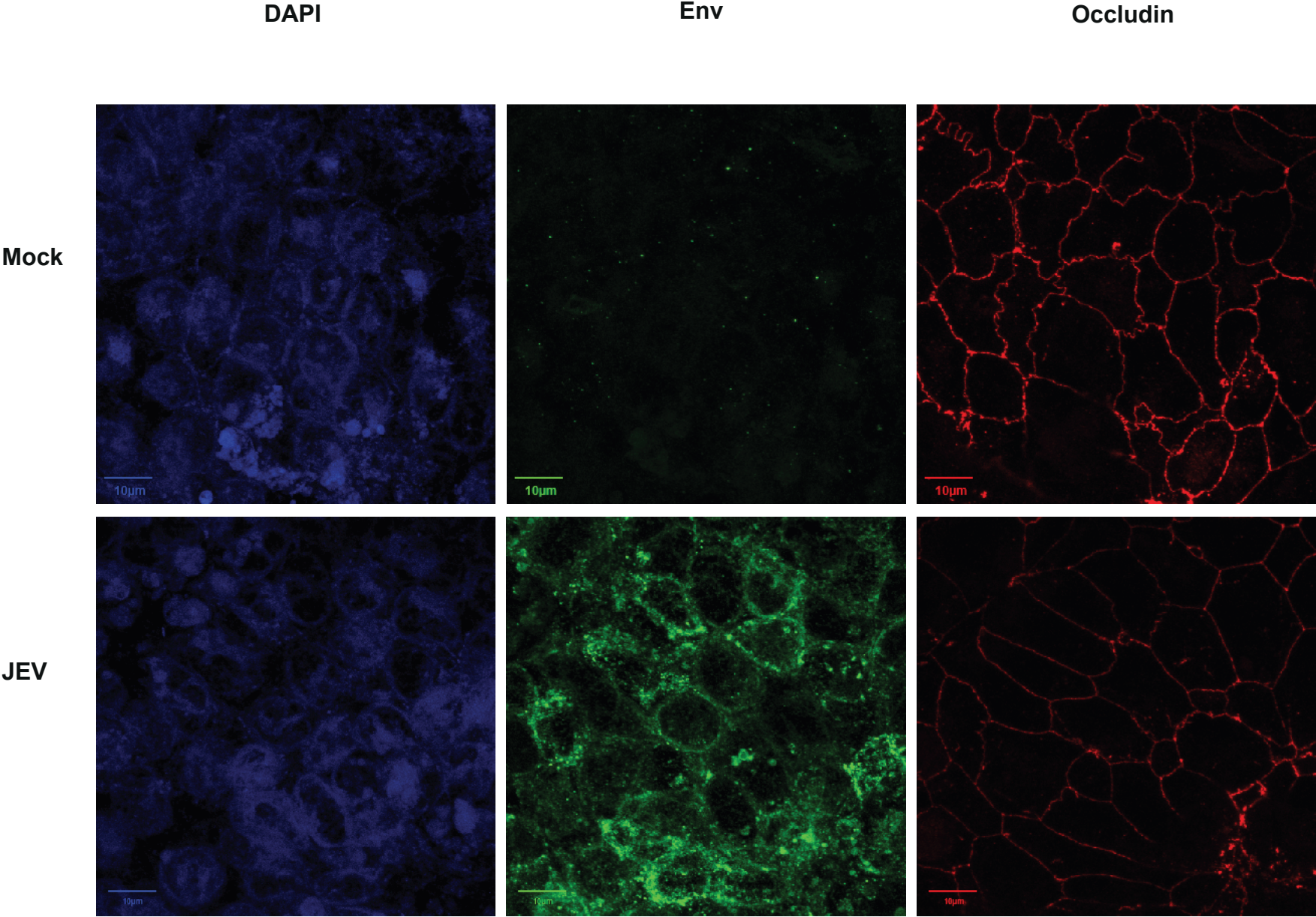

**Figure S2C:**

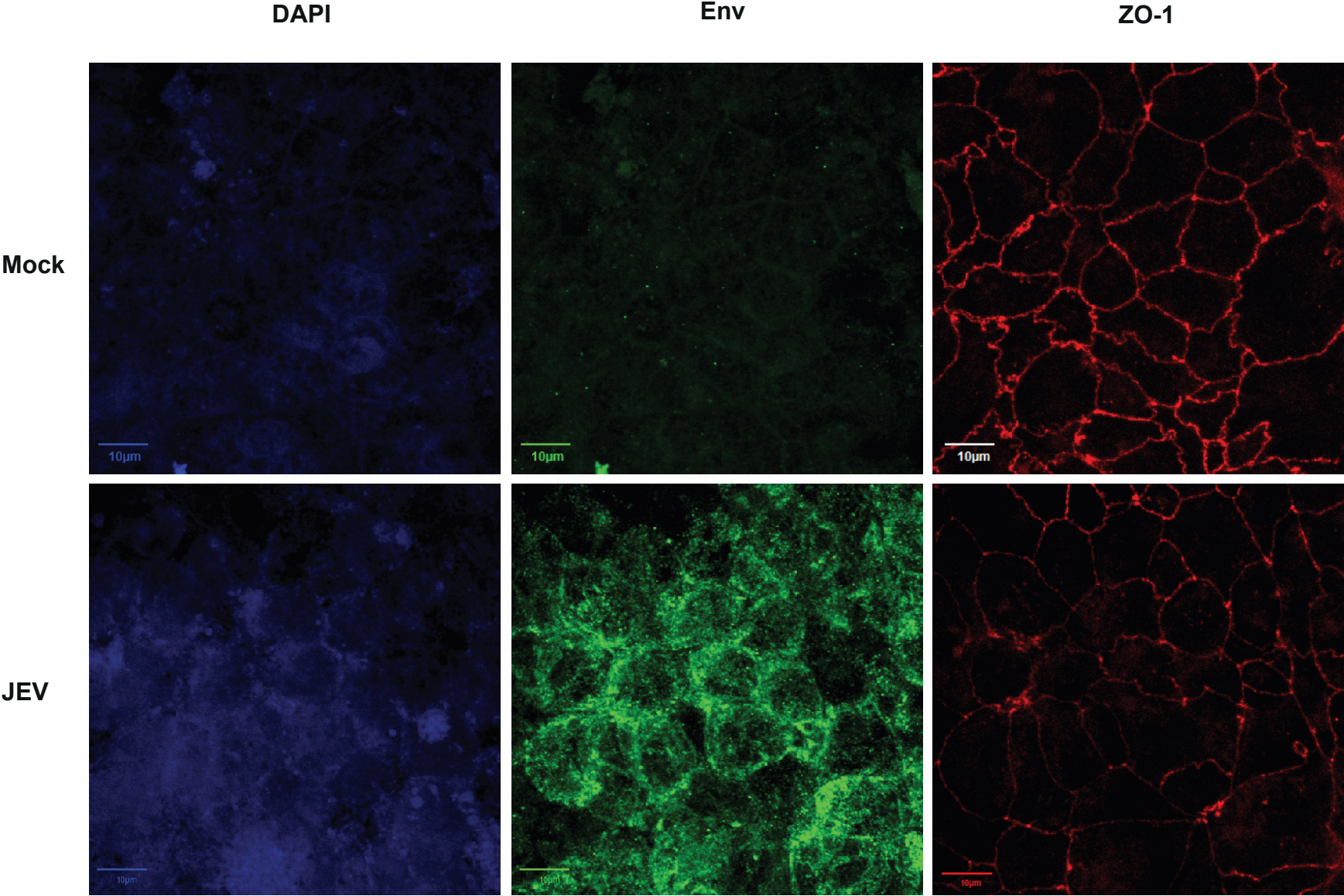

Supplement: Figure S2 — Localization of occludin and ZO-1 in JEV infected caco-2 cells. Caco-2 cells grown on trans-wells were infected with JEV and at 42 h p.i. cells were fixed and stained for JEV and β-catenin (A) occludin (B) or ZO-1 (C) as described in materials and methods. (PDF) [file pone.0069465.s002.pdf]

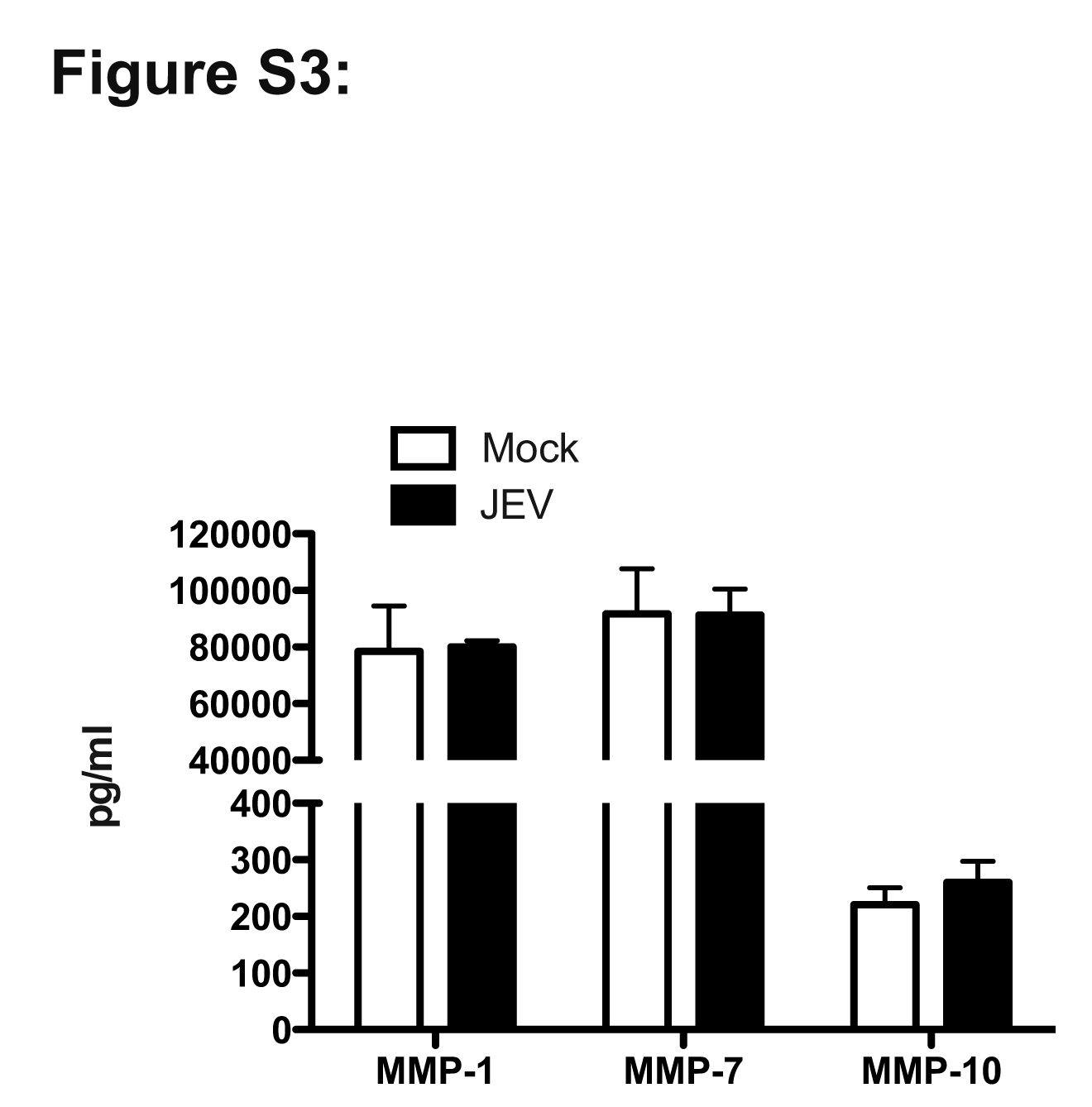

Supplement: Figure S3 — MMP levels in JEV-infected caco-2 supernatants. The amount of indicated MMPs in infected culture supernatants was measured by Luminex bead assays as described in materials and methods. The figures are representative of two experiments performed with two or more replicates. Error bars indicate mean with SEM. (TIF) [file pone.0069465.s003.tif]

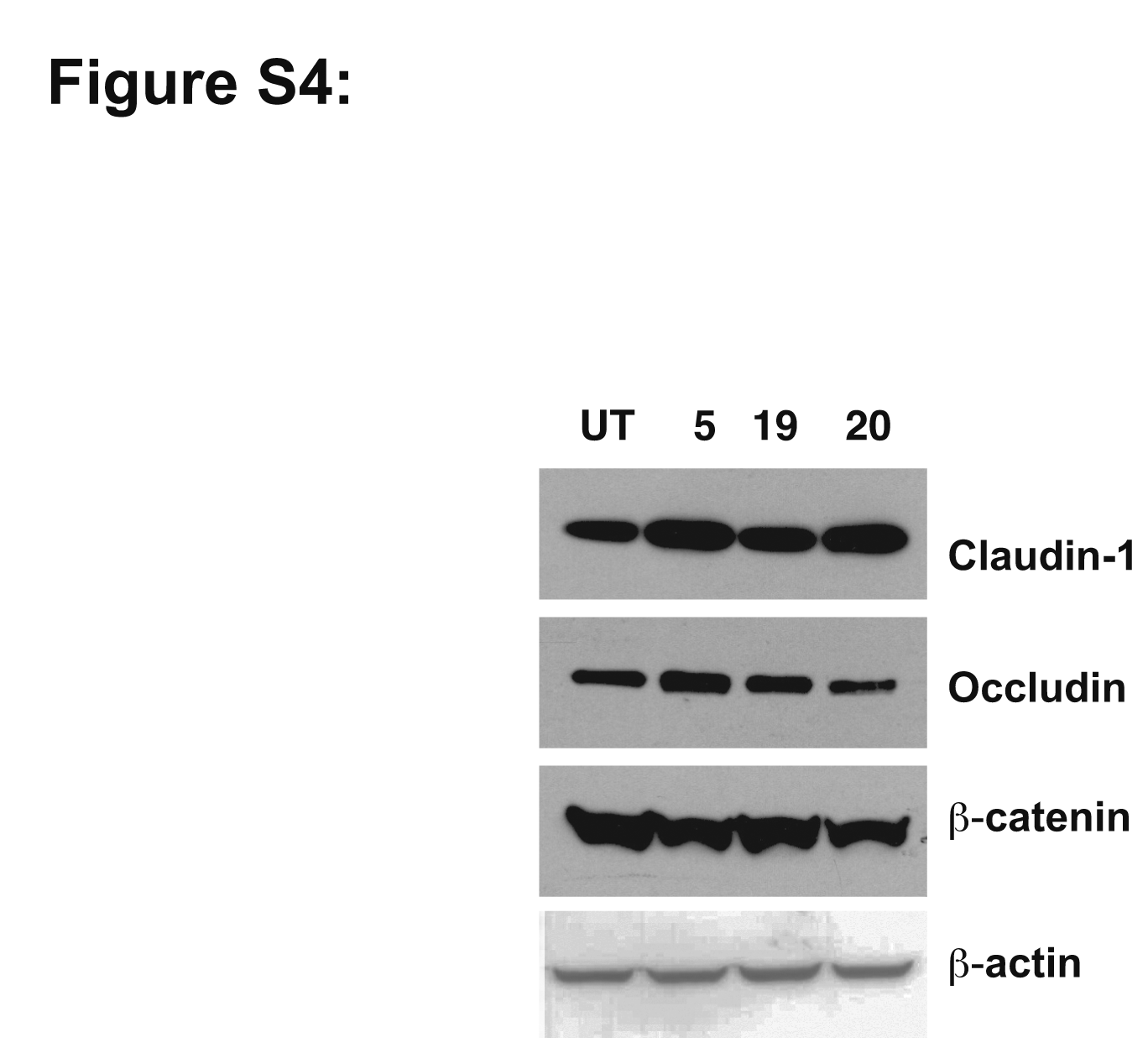

Supplement: Figure S4 — Levels of TJ proteins in Caco-2 clones expressing JEV-C. Cell lysates from untransfected (UT) or three stable clones (5, 19, and 20) expressing JEV-C were analyzed by western blot analysis for the indicated junctional proteins. (TIF) [file pone.0069465.s004.tif]

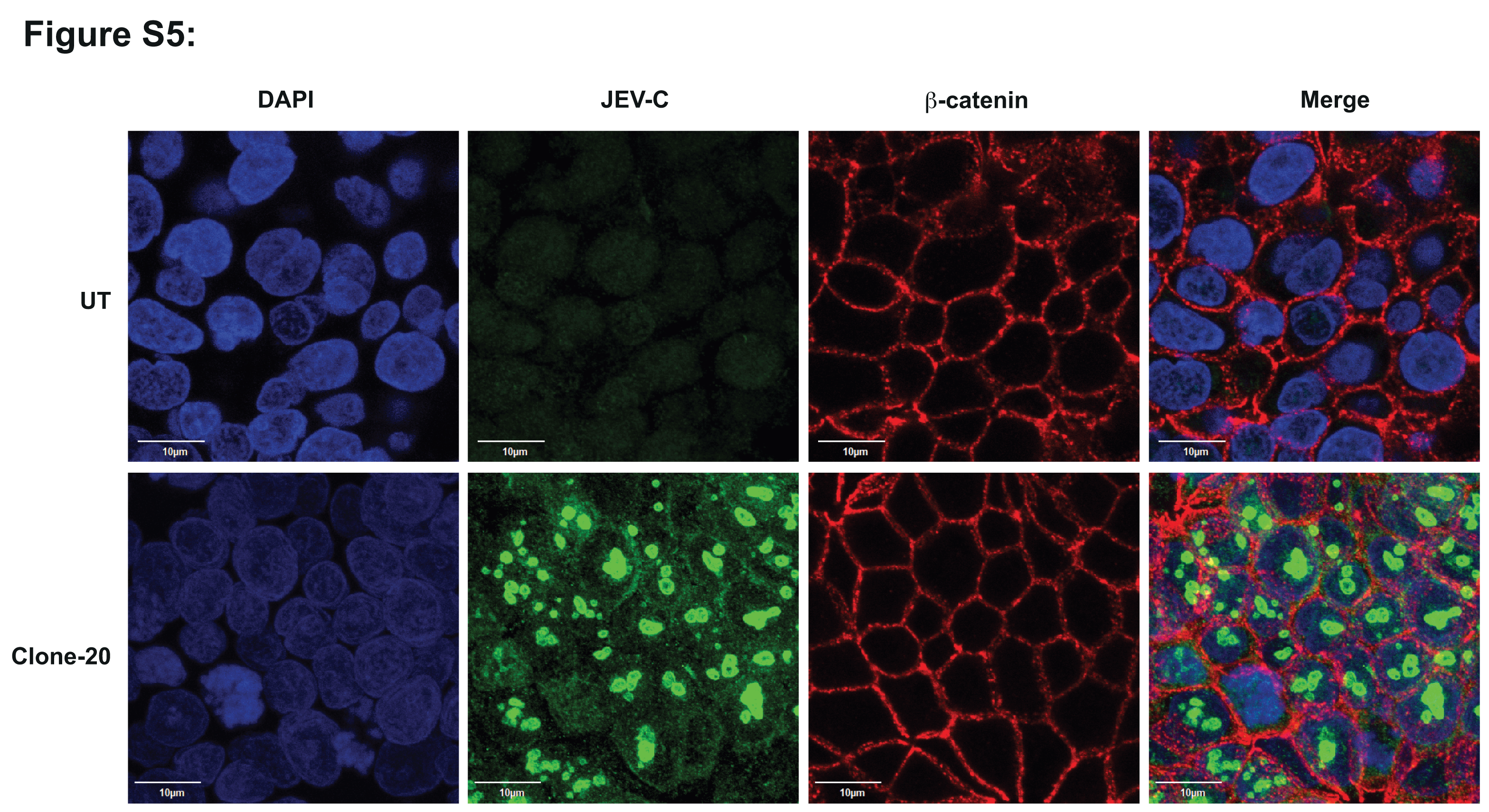

Supplement: Figure S5 — JEV-C expression does not affect β-catenin localization. Caco-2-C cells grown on trans-wells were fixed and stained for JEV-C (anti-His) and β-catenin as described in materials and methods. (TIF) [file pone.0069465.s005.tif]
