# Supplementary material for: Japanese Encephalitis Virus Disrupts Cell-Cell Junctions and Affects the Epithelial Permeability Barrier Functions
Source: PLoS One. 2013 Jul 24;8(7):e69465. doi: 10.1371/journal.pone.0069465 (PMC3722119; doi:10.1371/journal.pone.0069465)
Supplement: Table S1 — Sequence of forward and reverse primer (5′-3′) used in qRT-PCR in this study. (DOCX) [file pone.0069465.s006.docx]

Table S1: Sequence of primers and probes used in real-time PCR in this study

| **Name of the gene** | **Primer sequence (5’-3’)** |
| --- | --- |
| IFIH1 (MDA-5) | F: GTGATTCAGGCACCATGGGA  R: GGCTGGGCAACTTCCATTTG |
| IFI27 (ISG-12) | F: CCACGGAATTAACCCGAGCA  R: ATGGCCACAACTCCTCCAATC |
| DDX58 (RIG-I) | F: GGTATAGAGTTACAGGCATTTC  R: TTGTTTACTAGTGTTGTGGC |
| OAS-1 | F: ATTGTAAGAAGAAGCTTGGG  R: CAGAGTTGCTGGTAGTTTATG |
| CCL-5 (RANTES) | F: CTGCTTTGCCTACATTGCCC  R: CACACTTGGCGGTTCTTTCG |
| CXCL-10 (IP-10) | F: GTGGATGTTCTGACCCTGCT  R: GGAGGATGGCAGTGGAAGTC |
| DDIT3 (CHOP) | Assay ID: 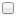Hs99999172_m1 (Life technologies) |
| ATF-3 | F: AGAAAGAGTCGGAGAAGC  R: TGAAGGTTGAGCATGTATATC |
| GAPDH | F: TGTGTCCGTCGTGGATCTGA  R: CCTGCTTCACCACCTTCTTGA  Probe: FAM-CCGCCTGGAGAAACCTGCCAAGTATG-TAMRA |
| Actin | F: ACCGAGCGCGGCTACAG  R: CTTAATGTCACGCACGATTTCC  Probe: FAM-TTCACCACCACGGCCGAGC-TAMRA |
