# Supplementary material for: Japanese Encephalitis Virus Disrupts Cell-Cell Junctions and Affects the Epithelial Permeability Barrier Functions
Source: PLoS One. 2013 Jul 24;8(7):e69465. doi: 10.1371/journal.pone.0069465 (PMC3722119; doi:10.1371/journal.pone.0069465)
Supplement: Methods S1 — (DOCX) [file pone.0069465.s007.docx]

**Supplementary methods**

Cell viability assays

For trypan blue exclusion, cells were trypsinized at 48 h pi and stained with trypan blue. Viable and dead cells were counted on hemocytometer. For cytotoxicity assays, cells were infected as described above and cytotoxicity was assessed by using lactate dehydrogenase release assay in the supernatants using CytoTox 96 Non-Radioactive Cytotoxicity Assay kit as per the manufacturer’s instructions (Promega catalog no. G1780).
